# Comparative Effectiveness and Safety of Amlodipine, Telmisartan, and Chlorthalidone in Newly Diagnosed Hypertensive Indian Adults

**DOI:** 10.7759/cureus.88272

**Published:** 2025-07-18

**Authors:** Mohammed Danish Anwar, Veena Verma, Sameer Gulati

**Affiliations:** 1 Department of Pharmacology, Atal Bihari Vajpayee Institute of Medical Sciences and Dr. Ram Manohar Lohia Hospital, New Delhi, IND; 2 Department of Pharmacology, Vardhman Mahavir Medical College and Safdarjung Hospital, New Delhi, IND; 3 Department of Medicine, Lady Hardinge Medical College, New Delhi, IND

**Keywords:** amlodipine, chlorthalidone, diastolic blood pressure, hypertension, systolic blood pressure, telmisartan

## Abstract

Objective: Amlodipine, telmisartan, and chlorthalidone are the most prescribed antihypertensive drugs in the Indian population. In this study, we compared the effectiveness and safety of these drugs in newly diagnosed hypertensive adults.

Methods: This was an open-label, prospective observational study in which we enrolled 99 newly diagnosed stage I hypertensive patients who were prescribed amlodipine (33 patients), telmisartan (33 patients), or chlorthalidone (33 patients) monotherapy. The primary endpoint was to compare the changes in systolic blood pressure (SBP) and diastolic blood pressure (DBP) at days 15, 30, 45, 60, and 90 from baseline among the three treatment groups. Secondary endpoints include incidence of adverse drug reactions (ADRs); changes in body weight, blood glucose, and lipid profile; and kidney function test on day 90 compared to baseline.

Results: All three groups showed a significant decrease in SBP and DBP on follow-up visits in comparison to baseline (p < 0.01), and the target BP was achieved by day 30. The incidence of ADRs was found to be lower in the telmisartan group (three) compared to the amlodipine group (five) and the chlorthalidone group (nine). The chlorthalidone group showed a significant increase in blood glucose and lipid levels (p < 0.05), whereas the telmisartan group showed a significant decrease in blood glucose and lipid levels (p < 0.05).

Conclusion: Telmisartan is more effective and safer as initial monotherapy followed by amlodipine than chlorthalidone.

## Introduction

Hypertension (HTN) is depicted as a persistent elevation of blood pressure (BP) that affects millions of people globally. In India, the overall prevalence of HTN is 30.7, with a higher prevalence in urban areas compared to rural areas [1]. As populations age and adopt more sedentary lifestyles, the prevalence of HTN continues to rise [2]. As there is a continuum of risk for cardiovascular ailments with increasing BP, HTN is a major preventable risk factor for cardiovascular disease and stroke, leading to an increase in morbidity and mortality worldwide [3,4]. Early detection and effective lowering of BP with antihypertensive drugs (AHDs) have been shown to reduce morbidity and mortality associated with cardiovascular and cerebrovascular events resulting from HTN [5]. Joint National Committee VIII guidelines recommend angiotensin II converting enzyme inhibitors (ACEI), angiotensin II receptor blockers (ARB), calcium channel blockers, or thiazide diuretics as first-line medication in the management of HTN [6]. Amlodipine, telmisartan, and chlorthalidone are some of the most commonly prescribed and widely used medications for the treatment of HTN.

Several studies are available comparing the efficacy and safety of AHDs; however, most of these studies are on Western populations, with results being extrapolated for other populations, including the Indian population [7,8]. As the Indian population is different from the Western population in terms of race, ethnicity, and food habits, their response to AHDs might be different from that of their Western counterpart [9]. Furthermore, to the best of our knowledge, there is a paucity of information on the safety and efficacy of antihypertensive medications in the Indian population, particularly when comparing the effectiveness of three distinct classes of antihypertensive medications at the same time [10]. Additionally, the majority of comparisons between antihypertensive medications are limited to two classes. Thus, it is crucial to generate a substantial amount of data regarding the safety and effectiveness of antihypertensive medications in the Indian population. This will help with the future development of Indian HTN guidelines. Therefore, the present study is planned to compare the effectiveness of telmisartan, amlodipine, and chlorthalidone as initial monotherapy in patients with stage I HTN and to compare their effects on lipid profile, blood glucose, renal parameters, body weight, and adverse drug reactions (ADRs).

## Materials and methods

Study design

It was an open-label prospective observational study that evaluated the effect of amlodipine, telmisartan, and chlorthalidone on systolic blood pressure (SBP) and diastolic blood pressure (DBP) in newly diagnosed stage I hypertensive patients conducted over a period of 18 months. The study was carried out in a tertiary care hospital after being approved by the Institutional Ethics Committee of Vardhman Mahavir Medical College (VMMC) and Safdarjung Hospital (S. no. IEC/VMMC/SJH/Thesis/2019-10/223) in accordance with the local regulations. Patients were enrolled after giving written informed consent.

Duration of study

The duration of the study was 18 months. It started from October 2019 to March 2021.

Selection of patients

**Figure 1:**
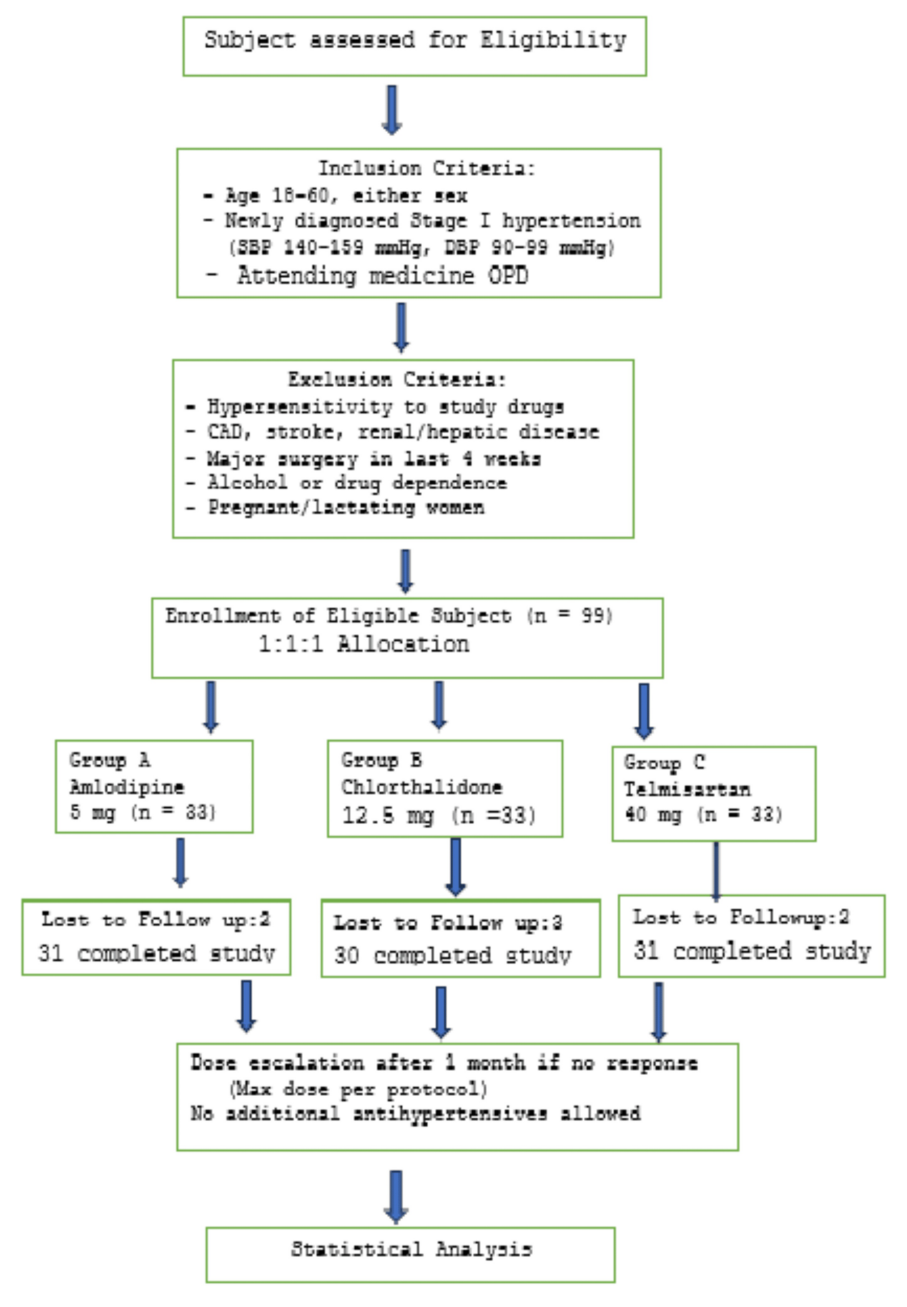
Flow diagram of methodology

A total of 99 subjects were enrolled in the study using the convenience sampling method due to the limited timeframe and the COVID-19 outbreak at that time. Using Cochran's formula with finite population adjustment, the sample size was determined. A study by Goyal et al. was referred to for estimating sample size [11]. The inclusion criteria include subjects of either sex who were 18-60 years of age, attending the medicine outpatient department, and newly diagnosed with stage I HTN according to Joint National Committee VIII guidelines (SBP 140-159 mmHg and DBP 90-99 mmHg) [6]. After initial screening, patients with a history of hypersensitivity to any of the interventional drugs, coronary artery disease, cerebrovascular incidents, renal or hepatic disease, major surgery within four weeks, alcohol or drug dependence, or pregnant or lactating women were excluded. All the required 99 participants were equally assigned (1:1:1) into three groups. Two participants were lost to follow-up visits in each of the amlodipine and telmisartan groups, while three were in the chlorthalidone group. Patients were assigned to receive amlodipine (5 mg) in group A, chlorthalidone (12.5 mg) in group B, and telmisartan (40 mg) in group C. If no response was seen after one month, then the dose was increased to the maximum dose for that drug. The choice of the AHD and dosage was at the discretion of the treating physician. Any additional antihypertensive medication precluded the subject from continuing in the study (Figure 1).

Outcome measures

The primary outcome is to compare the changes in SBP and DBP from baseline on days 15, 30, 45, 60, and 90 within the group and among the three treatment groups.

The secondary outcomes include the incidence of adverse events (AEs), changes in body weight, serum levels of fasting blood glucose, postprandial glucose, high-density lipoprotein (HDL), low-density lipoprotein (LDL), very low-density lipoprotein (VLDL), total cholesterol (TC) and triglycerides (TG), sodium (Na+), potassium (K+), creatinine, and blood urea nitrogen (BUN) on day 90 in comparison to baseline.

Data and blood sample collection

The patient’s SBP and DBP were measured at baseline and at days 15, 30, 45, 60, and 90 in the sitting position using a standardized procedure [12]. At the time of screening, detailed history and thorough clinical examination findings were recorded in the CRF. Blood samples were collected from the patients at baseline before starting the therapy and at day 90. Blood samples were centrifuged for serum separation and examined using enzyme-linked immunosorbent assay techniques specific to the measurement.

Statistical analysis

The data were entered in an MS Excel spreadsheet (Microsoft Corporation, Redmond, WA), and analysis was done using Statistical Package for Social Sciences version 23.0 (IBM Corp., Armonk, NY). Categorical variables were presented in numbers and percentages, and continuous variables were presented as mean ± standard deviation (SD). Normality of data was tested by using the Kolmogorov-Smirnov test. The reduction in SBP and DBP within each group was analyzed using the repeated measures analysis of variance (ANOVA), followed by Tukey's post hoc test. An unpaired t-test was used to analyze the primary outcome between the groups. The secondary outcome parameters were analyzed with a paired t-test within the group. Qualitative variables were compared using the chi-square test, and p < 0.05 was considered significant.

## Results

Demographic characteristics and baseline parameters of all three groups are shown in Table 1. Out of 33 patients, six in the amlodipine group, seven in the chlorthalidone group, and five in the telmisartan group required an increase in the drug dose to 10, 25, and 80 mg, respectively. During the study period, two patients were lost to follow-up visits in each of the amlodipine and telmisartan groups, while three were lost in the chlorthalidone group, so there were 31, 30, and 31 patients in the amlodipine, chlorthalidone, and telmisartan groups, respectively. All groups were similarly matched, and statistically insignificant differences were observed in demographic characteristics in terms of age, weight, and sex, except for body mass index (BMI) at baseline. The test applied was one-way ANOVA for all parameters except sex, for which the chi-square test was used. Only BMI showed a statistically significant difference across the groups (p = 0.027). When comparing the BMIs of the treatment groups using Tukey's post hoc analysis, the difference between amlodipine and chlorthalidone is found to be statistically significant (p = 0.0203).

**Table 1:** Demographic characteristics and baseline parameters of study participants Values are given as mean ± SD for quantitative variables ANOVA: analysis of variance; BMI: body mass index; HSD: honestly significant difference; SD: standard deviation

Parameter	Amlodipine	Chlorthalidone	Telmisartan	Test used	p value
Age (year)	48.26 ± 8.01	47.17 ± 7.95	47.29 ± 8.90	One-way ANOVA	0.705
Weight (kg)	70.42 ± 9.33	73.90 ± 8.51	73.06 ± 8.63	One-way ANOVA	0.082
Sex					
Male	17 (54.8%)	18 (60%)	20 (64.5%)	Chi-square	0.739
Female	14 (45.2%)	12 (40%)	11 (35.5%)		
Height (cm)	165.10 ± 6.96	165.20 ± 6.15	166.10 ± 4.98	One-way ANOVA	0.608
BMI (kg/m^2^)	25.81 ± 2.74	27.03 ± 2.34	26.47 ± 2.72	One-way ANOVA and Tukey's HSD post hoc analysis	0.027

Effect of amlodipine, chlorthalidone, and telmisartan on SBP and DBP

At baseline, both SBP and DBP were comparable in all three groups (p > 0.05) (Table 2). Following treatment, a significant decrease in both SBP and DBP was observed in all three groups compared to baseline and also on subsequent visits, i.e., days 30, 45, 60, and 90. The target BP of <130/90 was achieved both with amlodipine (128.97 ± 8.03) and telmisartan (124.38 ± 9.72) on day 30. However, the percentage reduction in both SBP and DBP was greater with telmisartan (SBP: 18%; DBP: 13.37%) than with amlodipine (SBP: 15.2%; DBP: 10.40%). Similarly, in all subsequent visits, the percentage decrease in both SBP and DBP was observed to be greater with telmisartan, followed by amlodipine, and less with chlorthalidone. The test applied was a one-way ANOVA followed by a post hoc Tukey test.

**Table 2:** Effect of amlodipine, chlorthalidone, and telmisartan on SBP and DBP (mmHg) Values are given as mean ± SD for quantitative variables ^*^p < 0.01 and ^**^p < 0.001 within the group from baseline to different timepoints, using one-way ANOVA followed by post hoc Tukey test SBP: systolic blood pressure; DBP: diastolic blood pressure; SD: standard deviation; ANOVA: analysis of variance

Timeline	Amlodipine group		Chlorthalidone group		Telmisartan group	
	SBP	DBP	SBP	DBP	SBP	DBP
Baseline	152.07 ± 4.23	95.46 ± 2.23	152.20 ± 4.21	95.58 ± 2.03	151.69 ± 4.44	95.10 ± 1.87
Day 15	138.32 ± 8.10^**^	89.38 ± 3.40^**^	141.78 ± 5.76^*^	90.93 ± 2.79^*^	133.23 ± 9.25^**^	87.10 ± 3.10^**^
Reduction from baseline	13.75 (9.04%)	6.08 (6.37%)	10.42 (6.84%)	4.65 (4.86%)	18.46 (12.17%)	8 (8.41%)
Day 30	128.97 ± 8.03^**^	85.53 ± 2.97^**^	135.17 ± 7.17^**^	88.29 ± 3.01^**^	124.38 ± 9.72^**^	82.38 ± 4.40^**^
Reduction from baseline	23.1 (15.2%)	9.93 (10.40%)	17.03 (11.19%)	7.729 (7.62%)	27.31 (18%)	12.72 (13.37%)
Day 45	126.22 ± 3.99^**^	83.93 ± 1.99^**^	132.53 ± 4.35^**^	86.85 ± 2.27^**^	121.36 ± 4.56^**^	80.30 ± 3.50^**^
Reduction from baseline	25.85 (16.99%)	11.53 (12.07%)	19.67 (12.92%)	8.73 (9.13%)	30.33 (19.99%)	14.8 (15.56%)
Day 60	125.49 ± 3.90^**^	83.46 ± 2.16^**^	131.96 ± 4.32^**^	86.73 ± 2.17^**^	120.48 ± 4.48^**^	79.41 ± 3.57^**^
Reduction from baseline	26.58 (17.47%)	12 (12.57%)	20.24 (13.29%)	8.85 (9.26%)	31.21 (20.57%)	15.69 (16.49%)
Day 90	124.87 ± 3.52^**^	82.95 ± 2.26^**^	131.25 ± 4.37^**^	86.48 ± 2.17^**^	119.72 ± 4.43^**^	78.99 ± 3.78^**^
Reduction from baseline	27.2 (17.88%)	12.51 (13.10%)	20.95 (13.76%)	9.1 (9.52%)	31.97 (21.07%)	16.11 (16.94%)

For the in-between group comparison, an independent t-test was applied. Comparison of the effects of amlodipine and chlorthalidone on SBP and DBP is displayed in Table 3. The percentage decrease in both SBP and DBP from the baseline was seen to be greater in the amlodipine group than in the chlorthalidone group in all the visits. This reduction in BP was found to be significant from day 30 (p = 0.001) and very significant on days 45, 60, and 90 (p < 0.0001).

**Table 3:** Comparison of amlodipine and chlorthalidone effects on SBP and DBP (mmHg) Data are represented as mean ± SD for quantitative variables. p values are calculated using an independent Student's t-test SBP: systolic blood pressure; DBP: diastolic blood pressure; SD: standard deviation

Time line	Amlodipine		Chlorthalidone		p value	
	SBP	DBP	SBP	DBP	SBP	DBP
Baseline	152.07 ± 4.23	95.46 ± 2.23	152.20 ± 4.21	95.58 ± 2.03	0.905	0.851
15th day	138.32 ± 8.10	89.38 ± 3.40	141.78 ± 5.76	90.93 ± 2.79	0.060	0.057
30th day	128.97 ± 8.03	85.53 ± 2.97	135.17 ± 7.17	88.29 ± 3.01	0.001	0.001
45th day	126.22 ± 3.99	83.93 ± 1.99	132.53 ± 4.35	86.85 ± 2.27	<0.0001	<0.0001
60th day	125.49 ± 3.90	83.46 ± 2.16	131.96 ± 4.32	86.73 ± 2.17	<0.0001	<0.0001
90th day	124.87 ± 3.52	82.95 ± 2.26	131.25 ± 4.37	86.48 ± 2.17	<0.0001	<0.0001

Comparison of the effects of chlorthalidone and telmisartan on SBP and DBP is displayed in Table 4. When we compared chlorthalidone and telmisartan, the percentage reduction in both SBP and DBP was seen to be greater with the telmisartan group than the chlorthalidone group, which was found to be significant from day 15 until subsequent visits (p value on day 90 was <0.00001).

**Table 4:** Comparison of chlorthalidone and telmisartan effects on SBP and DBP (mmHg) Data are represented as mean ± SD for quantitative variables. p values are calculated using an independent Student's t-test SBP: systolic blood pressure; DBP: diastolic blood pressure; SD: standard deviation

Timeline	Chlorthalidone		Telmisartan		p value	
	SBP	DBP	SBP	DBP	SBP	DBP
Baseline	152.20 ± 4.21	95.58 ± 2.03	151.69 ± 4.44	95.10 ± 1.87	0.713	0.340
15th day	141.78 ± 5.76	90.93 ± 2.79	133.23 ± 9.25	87.10 ± 3.10	<0.0001	<0.0001
30th day	135.17 ± 7.17	88.29 ± 3.01	124.38 ± 9.72	82.38 ± 4.40	<0.0001	<0.0001
45th day	132.53 ± 4.35	86.85 ± 2.27	121.36 ± 4.56	80.30 ± 3.50	<0.0001	<0.0001
60th day	131.96 ± 4.32	86.73 ± 2.17	120.48 ± 4.48	79.41 ± 3.57	<0.0001	<0.0001
90th day	131.25 ± 4.37	86.48 ± 2.17	119.72 ± 4.43	78.99 ± 3.78	<0.00001	<0.00001

Table 5 displays the comparison of the effects of amlodipine and telmisartan on SBP and DBP. The percentage reduction of SBP and DBP was observed to be greater with the telmisartan group than the amlodipine group, and the effect was significant from day 15 until subsequent visits (the p value on days 45, 60, and 90 was <0.001).

**Table 5:** Comparison of amlodipine and telmisartan effects on SBP and DBP (mmHg) Data are represented as mean ± SD for quantitative variables. p values are calculated using an independent Student's t-test SBP: systolic blood pressure; DBP: diastolic blood pressure; SD: standard deviation

Timeline	Amlodipine		Telmisartan		p value	
	SBP	DBP	SBP	DBP	SBP	DBP
Baseline	152.07 ± 4.23	95.46 ± 2.23	151.69 ± 4.44	95.10 ± 1.87	0.757	0.496
15th day	138.32 ± 8.10	89.38 ± 3.40	133.23 ± 9.25	87.10 ± 3.10	0.025	0.008
30th day	128.97 ± 8.03	85.53 ± 2.97	124.38 ± 9.72	82.38 ± 4.40	0.006	0.002
45th day	126.22 ± 3.99	83.93 ± 1.99	121.36 ± 4.56	80.30 ± 3.50	<0.001	<0.001
60th day	125.49 ± 3.90	83.46 ± 2.16	120.48 ± 4.48	79.41 ± 3.57	<0.001	<0.001
90th day	124.87 ± 3.52	82.95 ± 2.26	119.72 ± 4.43	78.99 ± 3.78	<0.001	<0.001

Effect of amlodipine, chlorthalidone, and telmisartan on metabolic parameters

With the amlodipine group, no significant changes in fasting blood sugar (FBS) and postprandial sugar (PPS) were observed on day 90 compared to baseline (p = 0.226 and 0.107). However, in the chlorthalidone group, a slight and significant increase in FBS (4.8%) and PPS (3.1%) at day 90 was found in comparison to baseline (p = 0.03 and 0.04). On the other hand, a significant (p < 0.05) decrease in FBS (8%) and PPS (4.58%) was observed at day 90 when compared to baseline in the telmisartan group. Similarly, on lipid profile parameters, with amlodipine, no significant changes were observed. However, compared to baseline, chlorthalidone caused a slight but significant increase in TC, LDL, VLDL, and TG levels and a decrease in HDL levels. On the other hand, the telmisartan group showed a slight decrease in TC, LDL, VLDL, and TG and an increase in HDL levels (p < 0.05). Amlodipine and telmisartan showed no changes in serum sodium, potassium, creatinine, and BUN. Although the chlorthalidone group showed a significant decrease in serum sodium and potassium at day 90 compared to baseline (p < 0.001), the values are within the normal range. No significant difference in body weight on day 90 from the baseline was observed in the three groups. The paired t-test was applied (Table 6).

**Table 6:** Effect of amlodipine, chlorthalidone, and telmisartan on metabolic parameters Values are given as mean ± SD for quantitative variables ^*^p value <0.05 and ^**^p value <0.01 from baseline to day 90 using paired t-test FBS: fasting blood sugar; PPS: postprandial sugar; TC: total cholesterol; HDL: high-density lipoprotein; LDL: low-density lipoprotein; VLDL: very low-density lipoprotein; TG: triglyceride; BUN: blood urea nitrogen

Metabolic parameters	Amlodipine		Chlorthalidone		Telmisartan	
	Baseline	Day 90	Baseline	Day 90	Baseline	Day 90
FBS (mg/dL)	98.65 ± 12.03	97.65 ± 9.68	99.40 ± 9.85	103.90 ± 14.63^*^	98.94 ± 12.34	91.35 ± 7.46^**^
PPS (mg/dL)	128.81 ± 14.81	127.42 ± 13.27	129.47 ± 13.39	132.80 ± 16.35^*^	130.84 ± 16.77	124.71 ± 12.47^**^
TC (mg/dL)	174.55 ± 13.28	173.81 ± 9.67	172.13 ± 12.78	178.13 ± 13.85^**^	176.26 ± 12.40	169.55 ± 12.29^**^
HDL (mg/dL)	44.74 ± 2.91	44.98 ± 2.40	45.73 ± 2.42	44.14 ± 3.08^*^	44.25 ± 3.27	45.33 ± 3.06^*^
LDL (mg/dL)	101.50 ± 11.29	100.79 ± 8.28	100.87 ± 10.20	107.22 ± 12.48^**^	104.43 ± 11.49	97.67 ± 11.41^**^
VLDL (mg/dL)	28.32 ± 5.29	28.07 ± 4.49	25.51 ± 5.04	26.72 ± 5.26^*^	27.68 ± 5.16	26.55 ± 4.76^*^
TG (mg/dL)	141.10 ± 26.70	138.97 ± 23.95	127.40 ± 24.85	134 ± 25.95^**^	136.61 ± 27.38	129.58 ± 24.90^**^
Sodium (mEq/L)	139.26 ± 2.83	139.23 ± 2.33	140.37 ± 2.91	137.67 ± 1.56^**^	139.39 ± 2.17	139.39 ± 2.03
Potassium (mEq/L)	4.61 ± 0.43	4.58 ± 0.44	4.56 ± 0.42	4.21 ± 0.42^**^	4.59 ± 0.44	4.60 ± 0.42
Creatinine (mg/dL)	0.84 ± 0.07	0.84 ± 0.08	0.86 ± 0.05	0.85 ± 0.05	0.86 ± 0.06	0.86 ± 0.05
BUN (mg/dL)	20.26 ± 3.13	20.35 ± 2.39	20.10 ± 2.60	20.10 ± 2.96	20.61 ± 2.91	20.61 ± 2.46
Weight (kg)	70.42 ± 9.33	70.34 ± 9.21	73.90 ± 8.51	73.52 ± 7.52	73.06 ± 8.63	72.94 ± 8.42

For in-between group analysis of metabolic parameters, an independent t-test was utilized. At day 90, the telmisartan group had the lowest glucose levels, especially when compared with the chlorthalidone group. While no significant difference in the lipid profile parameter was seen between the amlodipine and telmisartan groups, the telmisartan group, in comparison to the chlorthalidone group, showed a significant decrease in TC and LDL values. Among electrolytes, sodium and potassium were observed to be significantly lower in the chlorthalidone group than in the other two groups. For in-between group analysis of metabolic parameters, an independent t-test was utilized (Table 7).

**Table 7:** In-between group comparison of metabolic parameters showing p value p values are calculated using an independent t-test FBS: fasting blood sugar; PPS: postprandial sugar; HDL: high-density lipoprotein; LDL: low-density lipoprotein; VLDL: very low-density lipoprotein; BUN: blood urea nitrogen

Metabolic parameters	Amlodipine vs. chlorthalidone		Amlodipine vs. telmisartan		Telmisartan vs. chlorthalidone	
	Baseline	Day 90	Baseline	Day 90	Baseline	Day 90
FBS	0.702	0.126	0.773	0.008	0.988	0.001
PPS	0.573	0.161	0.741	0.269	0.988	0.047
Total cholesterol	0.281	0.206	0.597	0.135	0.076	0.013
LDL	0.925	0.024	0.176	0.223	0.103	0.003
VLDL	0.034	0.269	0.612	0.197	0.109	0.894
HDL	0.154	0.241	0.597	0.620	0.117	0.137
Triglycerides	0.036	0.440	0.512	0.123	0.177	0.500
Sodium	0.133	0.006	0.722	0.765	0.161	0.001
Potassium	0.649	0.001	0.861	0.860	0.658	0.001
Creatinine	0.415	0.885	0.496	0.838	0.857	0.385
BUN	0.760	0.712	0.734	0.593	0.513	0.646
Body weight	0.134	0.154	0.251	0.251	0.718	0.845

The overall incidence of AEs in all three groups was found to be low and of mild intensity. The number of patients reporting one AE was five (16.1%) with amlodipine, nine (30.0%) with chlorthalidone, and three (9.7%) with telmisartan. The chi-square test was applied. The incidence of ADRs was found to be significantly less in the telmisartan group (p = 0.046) when compared to the chlorthalidone group (Table 8).

**Table 8:** The number of adverse events reported with amlodipine, chlorthalidone, and telmisartan p value are calculated using chi-square test across amlodipine, chlorthalidone, and telmisartan groups

Parameters	Amlodipine	Chlorthalidone	Telmisartan	Chi-square value	p value
Nausea	1	2	0	-	-
Headache	1	4	1		
Dizziness	0	1	1		
Fatigue	1	2	1		
Constipation	2	0	0		
Total	5	9	3	7.93	0.44

## Discussion

According to recent WHO guidelines, individuals with a diagnosis of HTN and a BP measurement of 140 mmHg or above should be started on pharmacological antihypertensive therapy as soon as possible. However, if a patient has a history of cardiovascular disease, the threshold might be lowered to 130 mmHg [13]. Most HTN guidelines recommend any primary agent among the first-line AHD classes; however, details about the specific drugs are not given. Therefore, the uncertainty still remains about the most effective approach for the management of HTN [14,15]. Furthermore, not all drugs from the same class have the same level of antihypertensive potency, so their selection could potentially affect the probability of achieving BP control [16]. Considering the aforementioned explanations, it would be crucial to understand the antihypertensive effect of the most frequently used drugs, adjusted according to the most relevant clinical variables, as well as the characteristics related to better or worse treatment response. This knowledge would potentially help the clinician to choose the most appropriate treatment, since the response to a specific drug in a specific population could be better predicted. The present study was planned with the aim of comparing the efficacy and safety of telmisartan, amlodipine, and chlorthalidone as initial therapy in patients with stage 1 HTN, and also to evaluate their effects on lipid profile and blood glucose.

The primary endpoint in our study was to assess any change in the SBP and DBP from the baseline after treatment with amlodipine (5 mg once daily, OD), chlorthalidone (12.5 mg OD), and telmisartan (40 mg OD). In our study, all three groups, i.e., amlodipine, chlorthalidone, and telmisartan, showed a significant decrease in mean SBP and DBP in comparison to baseline at days 15, 30, 30,45, 60, and 90. However, the percentage decrease in SBP and DBP was found to be greater with the telmisartan group than with the amlodipine group and less with the chlorthalidone group in all the assessment visits. Tripathi et al. and Humagain and Koju also reported similar findings [17,18]. The target BP of <130/90 was achieved both with amlodipine (128.97 ± 8.03) and telmisartan (124.38 ± 9.72) on day 30 but not with chlorthalidone (131.25 ± 4.37). MacMahon et al. reported that a reduction in DBP of 5 mmHg is associated with a reduction of 21% in the incidence of congenital heart disease and at least 34% in the incidence of stroke [19]. Therefore, the significant difference, especially in decreasing DBP among the three drug groups seen in our study, may be of some clinical value.

Suthar et al. reported significant control in BP (p = 0.03) in patients receiving ARB as monotherapy when compared with other AHDs. A few side effects, like headache, dizziness, fatigue, and nausea, were reported after treatment. ARBs are considered a good option for those patients who are not able to take ACEIs [20]. Similarly, another study reported that ARBs are relatively safe, efficacious, and superior AHDs recommended for BP control. ARBs have unique renoprotective properties and are first-line antihypertensive agents in patients with impaired fasting glucose [21].

 In the present study, we have used chlorthalidone, not hydrochlorothiazide, in the thiazide class of AHDs. Due to its longer duration of action with significant reduction in BP and cardiovascular events with lesser metabolic disturbances, chlorthalidone has emerged as the low-dose diuretic of choice in the treatment of HTN [22]. The reductions in cardiovascular events have been noted with chlorthalidone in the Antihypertensive and Lipid-Lowering Treatment to Prevent Heart Attack Trial [23]. The antihypertensive effect of chlorthalidone in our study is in line with previous studies [24]. However, in comparison to amlodipine and telmisartan, the decrease in BP with chlorthalidone was found to be less.

In the present study, the amlodipine group showed no change in FBS, PPS, lipid profile, and serum electrolyte levels after 12 weeks of treatment. Similar findings have been observed in previous studies [25]. However, a slight increase in FBS (4.8%) and PPS (3.1%) was seen in the chlorthalidone group. On the other hand, the telmisartan group demonstrated a statistically significant decrease in FBS (8.0%) and PPS (4.58%) on day 90, but its clinical relevance is difficult to define. Tripathi et al. also reported comparable findings and concluded that telmisartan considerably lowered blood sugar levels and lipid variables, particularly in hyperglycemic and hyperlipidemic individuals [17].

Minimal increase in TC, LDL, VLDL, and TG values was seen with chlorthalidone compared to baseline in our study. Although the change in lipid profile parameters was significant, the values are within the normal range. A similar result is noted in the previous studies [26,27]. Telmisartan showed a favorable, albeit minimal, significant change in TC, LDL, VLDL, and TG levels. The beneficial effect of telmisartan on metabolic parameters was also reported in other studies [28]. The effect of telmisartan on lipid and glucose metabolism may possibly be explained by its high lipophilicity and its peroxisome proliferator-activated receptor gamma (PPAR) modulating effect [29,30].

Therefore, in the present study, telmisartan was found to be more effective than amlodipine and chlorthalidone in controlling BP. It showed a favorable effect on blood sugar and lipid profile, with no effect on serum electrolytes, and was reported to cause significantly fewer adverse effects compared to chlorthalidone. This unique action of telmisartan on PPAR leads to favorable effects on lipid and carbohydrate metabolism, which is independent of its BP-lowering effect.

 Strengths and limitations

In our study, we compared the effectiveness and safety of three commonly used first-line antihypertensive agents from different classes in Indian populations. Besides BP control, we also compared the effects of amlodipine, chlorthalidone, and telmisartan on lipid profile, blood glucose, kidney functions, and weight. Nevertheless, the study had a small sample size, a brief follow-up period, and was carried out in a single center without randomization.

## Conclusions

Based on the results of the present study, it can be concluded that telmisartan is more effective and safer than amlodipine and chlorthalidone in lowering BP. Given its favorable effects on metabolic parameters, telmisartan may be considered as a first-line agent for initial monotherapy of hypertensive patients, particularly those with dyslipidemia and diabetes. However, further studies with large sample sizes, including patients with comorbid conditions and for longer durations, are needed to substantiate these findings.
